# Tumor lysis syndrome in pediatric acute lymphoblastic leukemia at tertiary care center

**DOI:** 10.12669/pjms.35.4.715

**Published:** 2019

**Authors:** Bilqis Naeem, Khemchand N Moorani, Misbah Anjum, Uzma Imam

**Affiliations:** 1 Bilquis Naeem, MBBS, FCPS, FCPS, Assistant Professor, Pediatric Medicine, Departments of Pediatric Nephrology, National Institute of Child Health (NICH), Jinnah Sindh Medical University (JSMU) Karachi, Pakistan; 2 Prof. Khemchand N Moorani, FCPS, MCPS, MBBS, Departments of Pediatric Nephrology, National Institute of Child Health (NICH), Jinnah Sindh Medical University (JSMU) Karachi, Pakistan; 3 Misbah Anjum, MBBS, FCPS, Assistant Professor, Pediatric Medicine Pediatric Medical Unit III, National Institute of Child Health (NICH), Jinnah Sindh Medical University (JSMU) Karachi, Pakistan; 4 Uzma Imam MBBS, Senior Medical officer, Pediatric Oncology, National Institute of Child Health (NICH), Jinnah Sindh Medical University (JSMU) Karachi, Pakistan

**Keywords:** Tumor lysis syndrome, hyperuricemia, hyperphosphatemia, hypocalcemia, acute kidney injury

## Abstract

**Objectives:**

Tumor lysis syndrome (TLS) is common complication of acute lymphoblastic leukemia (ALL). It is characterized by presence of two or more of hyperkalemia, hyperuricemia, hyperphosphatemia and hypocalcemia. TLS may cause acute kidney injury (AKI), arrhythmias and seizures. Our objective was to determine the frequency of TLS and its biochemical abnormalities in children with ALL.

**Methods:**

A retrospective study on 91 children, aged 2-13 years with ALL was carried out in Nephrology and Oncology departments of National Institute of Child Health, Karachi from January 2016 to December 2017. Patients already received chemotherapy were excluded. Data including risk categories, immunophenotyping, laboratory parameters like complete blood picture, serum creatinine (SCr), potassium(K), calcium (Ca), phosphorus(P) and uric acid (UA) on day 0,3 and 7 after chemotherapy were collected. Data analyzed on SPSS using descriptive statistics. Independent t- test was applied to compare means and P- value<0.05 was taken as significant.

**Results:**

Ninety-one children with mean age of 6.39±3.08 years were studied. Male were 57% and 43% female. High risk ALL were 61.5%. Pre –BALL were 82.4% and 17.5% had T-cell ALL. All patients had anemia (hemoglobin7.69±2.66 g/dl) and thrombocytopenia (43.61± 18.6 x10^9^) where as hyperleukocytosis and blast cells were observed in 20.87% and 73.6% respectively. Comparing the biochemical parameters of ALL, the difference in SCr from D0 vs D3 (0.46±0.16 vs0.54± 0.35 and D7, 0.44±0.22) was significant (p=0.001). Similarly, difference in UA (D0, 4.12±2.40 vs D3, 3.82±1.73 and D7, 3.56±1.42), SP (D0, 4.24±1.34 vs D3, 4.61±1.76 and D7,4.13±1.07mg/dl)and for K (p=0.038) was significant. There was no difference in Ca from D0 vs D3 (0.092) and D7 (0.277). TLS was found in 62.6% children, it was chemotherapy induced in 72% and spontaneous in 28%. Clinical-TLS was observed in 14% and all CTLS had AKI. Hyperuricemia and hyperphosphatemia were the most common biochemical abnormalities in laboratory-TLS and CTLS.

**Conclusion:**

TLS was found in 62.6% despite preventive measures. Early recognition and treatment is essential to avoid morbidity and mortality.

## INTRODUCTION

Tumor lysis syndrome (TLS) is an oncological emergency resulting from massive lysis of malignant cells and clinically characterized by renal failure, seizures and cardiac arrhythmias that requires early recognition and management.^1^ It is a life-threatening condition with high morbidity and mortality.^2^ This lysis of malignant cells may occur spontaneously before treatment or after induction with chemotherapy and accordingly it is called spontaneous TLS and chemotherapy induced TLS.

The massive lysis of lymphoblastic cells releases intracellular metabolites like potassium (K), uric acid (UA), phosphate (P) and products of protein and purine metabolites into systemic circulation. These high effluxes of metabolites result in abnormal accumulation in the blood and exceed the capacity of renal clearance and results hyperkalemia, hyperuricemia, hyperphosphatemia and secondary hypocalcemia. Hyperuricemia is the initial and most common abnormality, induces acute renal injury by intra-renal uric acid crystallization and It aggravates precipitation of calcium phosphate crystals in the renal tubules. This crystal nephropathy causes inflammation, obstruction and renal tubular damage ultimately manifesting as acute kidney injury. Hyperkalemia may also be aggravated by crystal nephropathy induced AKI and may cause serious dysrhythmias and death. Hypocalcemia may lead to muscle cramps, tetany, seizures and dysrhythmias. There is concomitant rise of lactic dehydrogenase because of rapid cell turn over in ALL.^1^-^4^

AKI may occur in ALL in absence of TLS due to renal infiltration by blast cells, infections and volume depletion.^5^ According to Cairo and Bishop criteria, the laboratory TLS (LTLS), is present if >two of following abnormalities are present within three days before or up to seven days following chemotherapy, namely hyperuricemia (UA>8 mg/dl), hyperphosphatemia (P>6.5mg/dl), hyperkalemia (K>6 meq/L) and hypocalcemia (calcium<7 mg/dl). Clinical TLS (CTLS) is characterized by any one of end organ clinical features like acute kidney injury(elevated serum creatinine > 1.5 times the upper limit of normal and oliguria for 6 hours), features of leukostasis (seizures, intracranial bleed), cardiac arrhythmias and death.^1^,^5^ The risk of TLS depends upon multiple factors like, aggressive nature /type of leukemia, stage of disease at diagnosis, leukemic burden, lactate dehydrogenase (LDH)level and regimen of chemotherapy and its chemo sensitivity in addition to dehydration, pre-existing renal damage and sepsis.

More aggressive nature, rapidly proliferating tumor, with high leukemic burden and highly sensitive to drug treatment and high-risk patients are more likely to develop TLS.^3^-^5^ TLS can occur spontaneously in highly aggressive tumors like Burkitt’s lymphoma and acute lymphoblastic leukemia (ALL) but it can occur after treatment in less aggressive tumors.^5^-^7^

Acute lymphoblastic leukemia is the most common hematological malignancy and accounts for 75-80% of all childhood leukemia.^6^-^8^ ALL occurs in three out of 100,000 children in the world and in Pakistan it has been propagated around 3000 new cases per year.^9^

ALL is most aggressive malignancy associated with high mortality (11.5%-24%) in Pakistan.^10^,^11^ The primary treatment of ALL is cytoreduction by induction chemotherapy along with hyper hydration and strict monitoring for complications particularly TLS.^2^,^8^ The reported prevalence of TLS across the world in hematological malignancies varies from 20-42 %.^1^

Since TLS is an acute emergency so early identification of high-risk patients, appropriate prophylactic interventions like hyper hydration, use of allopurinol, rasburicase and strict monitoring for its clinical complications like arrhythmia, AKI and central nervous system (CNS) manifestations in leukemic patients undergoing chemotherapy may help to decrease frequency, severity of TLS and improve outcome.^2^,^4^,^5^

There are number of local studies on various aspects of ALL but very few on incidences of TLS in children.^7^,^8^,^10^,^11^

Our pediatric oncology unit is functioning for last 15 years with an average of 500 patients are registered per year and more than 50% among them are suffering from ALL. We want to share our retrospective data of last two years, focusing on this serious complication of ALL in children.

The objective of this study was to determine the frequency of TLS in children with ALL and various biochemical abnormalities in children with TLS.

## METHODS

This retrospective cross-sectional study was conducted on 91 children with ALL who were managed in the Department of Pediatric Oncology, National Institute of Child Health, Karachi, from January 2016 to December 2017.

### Operational Definitions

Laboratory TLS was defined when two or more of the following biochemical abnormalities were present (i) hyperuricemia (ii) hyperkalemia (iii) hyperphosphatemia (iv) hypocalcemia, Clinical TLS was diagnosed if child had abnormality in any one end organ (eg AKI, seizure and cardiac arrhythmias) along with lab TLS.

Acute kidney injury was defined if more than 50% rise in serum creatinine (SCr) from baseline or SCr was more than 1.5 time upper limit of normal.

### Risk stratification

Patients were categorized according to The National Cancer Institute (NCI) criteria into standard risk and high -risk groups, based on age at diagnosis, initial white blood count (WBC), central nervous system or testicular involvement .^12^

### Immunophenotyping

Patients were classified into precursor -B leukemia (pre-B Cell) and precursor T-cell leukemia (pre-T-Cell), based on flow cytometry and immunohistochemistry.

### TLS Prophylaxis

Tumor lysis prophylaxis with hyper hydration (125 ml/m^2^/hour)and allopurinol as inhibitor of UA synthesis (10 mg/kg/day in 3 divided doses) in cases with high UA levels was started at least 24 hours before and 4 days after chemotherapy.

### Inclusion and exclusion criteria

All patients aged 2-13 years hospitalized during the study period with the diagnosis of acute leukemia and managed in the Pediatric Oncology & Nephrology Department were studied. Patients who received initial chemotherapy at other institute and not underwent induction phase or refused chemotherapy were excluded.

Data collected included bio-data, anthropometry, various clinical and laboratory parameters from hospital record in the predesigned proforma. The laboratory parameters included complete blood picture, UA, P, Ca, SCr and K measured as baseline (day 0) then daily after initiation of chemotherapy till 7^th^ day. Estimated glomerular filtration rate (eGFR) was calculated using Schwartz formula from patient’s height and SCr^.13^Blasts cells(>5%) in peripheral smear, lactic dehydrogenase (LDH) level, type of ALL based on immunophenotyping and risk stratification group were also recorded. Ethical approval was taken from hospital ethical committee.

### Statistical Analysis

For numerical variables like age, S.Cr, S.K, mean±SD was used and categorical variables like gender, frequency was used. Data was entered in SPSS version 20. Independent t- test was used for comparison of means. P value <0.05 was considered as significant with confidence interval of 95%.

## RESULTS

Data of 91 patients were included in this study. Baseline characteristics of children with ALL are shown in Table-I. Fifty-two (57.14%) were male and 39 (42.92%) were female. The mean age±SD of study population were 6.39± 3.08 years with range of 2-13 years. Majority of patients (73, 80.21%) were below 10 years of age. The mean height and weight of patients with ALL were 110.89±18.95cm and16.74± 6.58 kg respectively. The mean body surface area was 0.699± 0.21 m^2^.Majority (82.41%) had Pre-B ALL and 16 (17.58%) had T-cell leukemia. On risk stratification, 56 (61.53%) patients were in high risk group whereas 35 (38.46%) in standard risk leukemia.

**Table I:** Baseline characteristics of in children with Acute Lymphoblastic Leukemia (n=91).

Variable	Number/mean±SD	Range/Percentage
Age (mean±SD years)	6.39±3.08	2-13
<10 years	73	80.21%
>10 years	18	19.78%
*Gender:* Male	52	57.14%
Female	39	42.85%
Weight (kg)	16.74±6.58	7-35
Height (cm)	110.89±18.95	74-157
Body surface area(m^2^)	0.7±0.21	0.17-1.6
*Hematological parameters*		
Hemoglobin(g/dl)	7.69±2.66	3.20-14.90
Mild anemia (< 11)	7	7.6%
Moderate (7-10	44	48.4%
Severe(<7)	40	43.9%
White blood count	37.27±13.69	.001-75.000
< 50x10^9^/L	56	61.5%
50-100x10^9^/L	15	16.4%
>100x10^9^/L	19	20.8%
Platelet count/ul (mean±SD)	4361.6±18606	0.40-105000
*Diagnosis*		
Peripheral smear (>5% blast cell)	77	84.6%
Bone marrow (>25% blast cells)	14	15.4%
Lactic dehydrogenase (U/L)	2499.3±3926.4	3.60-20295
*Immunophenotyping:*		
Pre-B-cell immunophenotype	75	82.4%
Pre-T-Cell immunophenotype	16	17.6%
*Risk category:*		
High risk	56	61.5%
Standard risk	35	38.5%

Hematological findings (Table-I) revealed that almost all patients had anemia(mean hemoglobin 7.69± 2.67 g/dl) and mean white blood counts was 37.27± 13.69x 10 9.Leukemic burden was very high (hyper leukocytosis >100x10 9/L) in 19 (20.87%), high (50-100x 10^9^/L) in 15(16.48%) and standard (wbc < 50x10^9^/L) in 56(61.53%). Similarly, all patients had thrombocytopenia with a mean platelet count 43.61± 18.6 x10^9^and majority (61%) had thrombocytopenia (<50x10^9^/L). Sixty-seven(74%) were diagnosed as ALL on the basis of presence of blasts cell in the peripheral smear and rest on bone marrow.

Base line biochemical parameters and renal functions in children with ALL are shown in Table-II; which shows that mean serum levels of UA, P and Ca were 4.12±2, 4.24±1.34, and 9.4±1.02 mg/dl respectively. The baseline means serum SCr and eGFR was 0.46±0.166 mg/dl and143.66±58ml/min/1.73 m^2^respectively.Table-II

**Table II:** Baseline biochemical parameters and renal functions in children with Acute Lymphoblastic Leukemia (n=91).

Parameter	Mean±SD	Range
Serum creatinine (mg/dl)	0.46±0.17	0.12-1.16
eGFR ml/min/1.73 m^2^	142.66±58	34-375
Serum uric acid (mg/dl)	4.12±2.0	1.4-10.9
Serum phosphate (mg/dl)	4.24±1.34	1.1-8.4
Serum potassium (meq/l)	4.1±0.72	2.3-7.7
Serum calcium (mg/dl)	9.4±1.02	6.4-11.7

eGFR: estimated glomerular filtration rate

Comparative changes in various biochemical parameters from base line (D0) to D3 and D7 of induction chemotherapy are shown in Table-III. These biochemical changes on 7^th^day showed that mean±SD levels of UA, P and Ca was3.56±1.42, 4.13±1.07and 9.16±0.84mg/dl respectively whereas serum K was 3.76±0.51meq/L. The mean serum Cr was 0.44±0.22 mg/dl. There was significant difference (p=0.001) in SCr fromD0(0.46±0.166 mg/dl) vs. D3(0.54±0.35mg/dl) and D7(0.44±0.22 mg/dl).There was significant difference (p=0.001) in mean levels of serum UA from D0(4.12±2.40 mg/dl) vs. D3(3.82±1.73 mg/dl) and D7(3.56±1.42 mg/dl).Similarly, there was significant difference(p=0.001) in S.P from D0(4.24±1.34 mg/dl) vs. D3(4.61±1.76 mg/dl) and D7(4.13±1.07mg/dl).There was significant difference (0.038) in K from D0(4.10±0.72 meq/L) to D4 (3.92±0.55 meq/L) only. There was no significant difference of mean serum Ca from D0 vs. D3 (0.092) and D7 (0.277).

**Table III:** Comparison of biochemical parameters in children with tumor lysis syndrome (n=91).

Days	S. creatinine (mg/dl)	Uric acid (mg/dl)	Phosphate (mg/dl)	Potassium (meq/L)	Calcium (mg/dl)
0	0.46±0.17	4.12±2.0	4.24±1.34	4.10±0.72	9.4±1.02
3	0.54±0.35*	3.82±1.73*	4.61±1.76*	3.97±0.52	9.28±1.2
7	0.44±0.22*	3.56±1.42*	4.13±1.07*	3.76±0.51	9.16±0.84

P- value <0.05

* Significant

Over all, TLS was found in 57 (62.6%) children with ALL either spontaneously or after induction chemotherapy. All patients fulfilled the criteria of LTLS whereas clinical TLS was found in 8 (14. 03%) cases. Though, AKI was found in 11 cases but three did not fulfil TLS criteria. However, cardiac arrhythmias and seizures were not observed in any case.

**Fig. 1:**
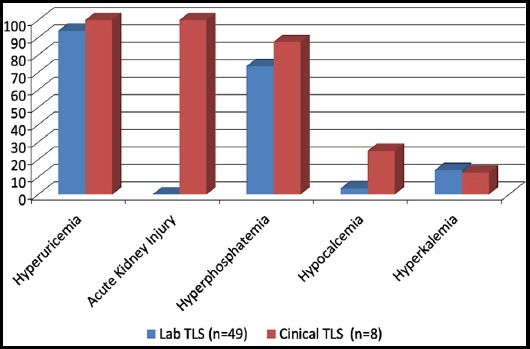
Comparative frequency of biochemical and renal abnormalities Tumor Lysis Syndrome (n=57).

Comparative frequency of biochemical and renal abnormalities are shown in Fig-1. Which shows that hyperuricemia and hyperphosphatemia were the most common abnormalities in both LTLS and CTLS and found in 82.4%vs 87.5% and 73.6% vs 87.5% respectively. Hyperkalemia and hypocalcemia were the least common abnormalities. All CTLS had AKI. Among patients with LTLS, 42 had two biochemical abnormalities, eight (14%) had three and two had four as defined by Cairo-Bishop criteria of LTS.

## DISCUSSION

TLS occurs in 20- 40% in hematological malignancies and is a serious problem associated with high morbidity and mortality in children with leukemia and lymphoma. So, it is important to recognize risk factors and to initiate preventive strategies.^2^-^4^

In our study, TLS was very common in children with ALL and LTLS was found in 62.6%and CTLS in 14% of cases. The reported prevalence varies across the world and our findings are higher than the reported figures, ranging from 25.5 to 39%.^14^-^17^ But our findings of TLS in 62.6% of cases are consistent with studies by Kedar A et. al and Darmon et al in which 70% and 63.8% of children with acute leukemia had TLS.^15^,^18^ However, high figures in our study may be due to the most common malignant condition associated with very high leukemic burden, hyperleukocytosis. The recent study from Ethiopia by Michu H et al has reported 29.5% of TLS in children with all types of childhood malignancies over a period of 6 months but our study duration was one year, and number of patients were also high (n=91) compared to 61 cases.^17^ In a study by Abdul-Basit HA et al showed the frequency of TLS in 45% which is slight lower than our study.^19^

In local studies on various aspects of childhood ALL, the reported frequency of TLS varies from 10-39%.^7^,^10^,^20^ This wide variation in range of incidence of TLS could be explained on lack of standard criteria for diagnosis of TLS, variation in prophylaxis protocol and patients cohort, age difference and stage of disease, delayed diagnosis, delay in initiation of chemotherapy and lack of use of effective preventive therapy(like rasburicase) particularly in developing countries.^14^,^15^,^21^

Considering the biochemical abnormalities in children with TLS, most common abnormality was hyperuricemia (59.3%) followed by hyperphosphatemia (53.8%). Though, our patients were receiving allopurinol along with intensive hydration, but it seems that elimination of circulating uric acid was not enough in the face of rapid production. Second most common biochemical abnormality was hyperphosphatemia. In study by Bagshi et al. hyperphosphatemia was the most common (95%) abnormality and all patients with hyperphosphatemia had AKI and 81% had hyperphosphatemia even before chemotherapy.^22^

Two or more than two metabolic abnormalities were found in 73 %of cases with LTLS whereas in CTLS (8, 14%) more than 87.5% children had two biochemical abnormalities suggesting that all those who developed AKI after cytoreduction therapy was result of hyperuricemia and hyperphosphatemia. Almost all children with high risk stratification (n=56) and pre-B cell leukemia had developed TLS in our study. Similar prevalence of TLS in high risk ALL (all 36 patients) and pre-B cell phenotyping has been reported by Mansoor et al and Saeed F et al (37%) recently.^20^,^23^

The prevalence of AKI in different studies varies from 5-40% but more recent reports suggest that incidence of AKI varies from14.2-75% depending upon type of patients more in aggressive lymphomas and leukemias. Khalil and colleagues reported AKI in 31.8% in adults with lymphoma whereas Waseem et al. has shown AKI in 40% cases.^24^,^25^ Hyperkalemia was found in 9 patients (9.9%) and hypocalcemia in 4 patients (4.4%).

On comparison of serum UA, P on D0 with D7post-chemotherapy, we found significantly higher than from baseline suggesting that induction chemotherapy is the main risk factor for developing TLS. One most important consideration of our study was that majority (61.5%) of patients had high risk category and pre-B ALL on risk stratification and these patients are more likely to developed TLS due to hyperleukocytosis. In addition, high LDH levels (2499.3±3926.4U/L) in our patients may be an indicator of TLS has also have contributed for development of TLS in this high-risk group.^25^

## CONCLUSION

Tumor Lysis Syndrome was frequent complication in pediatric ALL. Despite of all preventive measures like hyperhydration and use of allopurinol in our study, we found high frequency (62%) of TLS. We suggest more vigilant assessment and monitoring to recognize and treat those patients who are at risk of TLS. Our study’s strength was that it has focused a single and most common childhood malignancy compared to inclusion of multiple hematological malignancies in most studies. Main limitation is retrospective study from single center. A large multicenter study is needed to establish exact incidence in pediatric hematological malignancies.
